# Assessing environmental impact of genetically modified seeds in Brazilian agriculture

**DOI:** 10.3389/fbioe.2022.977793

**Published:** 2022-08-30

**Authors:** Renato Nunes de Lima Seixas, José Maria Ferreira Jardim da Silveira, Vinícius Eduardo Ferrari

**Affiliations:** ^1^ Economics Department, Federal University of Espírito Santo, Vitória, Espírito Santo, Brazil; ^2^ Institute of Economics, State University of Campinas, Campinas, São Paulo, Brazil; ^3^ Post-Graduation Program in Sustainability, Center for Economics and Administration, Pontifical Catholic University of Campinas, Campinas, São Paulo, Brazil

**Keywords:** environmental impact, transgenic seeds, stacked genes, pesticides, new breeding technologies, CRISPR

## Abstract

Genetically modified (GM) seeds have had relevant impacts on worldwide agriculture, even with a limited number of essential traits launched in the markets. The focus on platforms crops has favored the combination of traditional breeding, GM insertion, and diffusion in agriculture. One of the remarkable features of the GM traits has been the close link with pest and weed control systems. We investigate the environmental effects due to pesticides for two different GM seeds: insect resistant (IR) cotton and herbicide tolerant (HT) soybeans in a particular period of Brazilian agriculture, 2009–2013. We use a dataset on commercial farms' use of pesticides and biotechnology in Brazil to document environmental effects of GM traits. We explore within farm variation for farmers planting conventional and GM seeds to identify the effect of adoption on the environmental impact of pesticides measured as the quantity of active ingredients of chemicals and the Environmental Impact Quotient (EIQ) index. The findings show that the IR trait reduces application of insecticides by 22% and the associated environmental impact by 20% the environmental impact of insecticides. However, for HT traits, we find that application of herbicides increases by 55.8% and the associated environmental impact by 44.4%, showing a significant increase in the EIQ. The HT results are driven by an increase of less toxic herbicides elevenfold larger than the decrease in less toxic ones, which we interpret as evidence of weak substitutability between herbicides of different toxicity levels. Addressing what happened in the last decade, the paper also presents a view of the transformations in GM usage in Brazil, focusing on the considerable success in adopting stacked genes. Future perspectives point to a more diversified menu of technologies, crops, and adopting countries, going beyond platform crops and more prominent agriculture exporters.

## 1 Introduction

GM seeds have been considered one of the major technological innovations for agricultural systems and have been promoted as an effective tool for controlling agricultural pests and expanding food supply. Their relevance can also be measured by the wide span of controversial issues that have been raised in the related literature since their introduction. Those involve intellectual property rights over organisms, productivity effects, economic returns, consumer safety, welfare and income distribution, and environmental effects (Graff et al., 2003; Qaim, 2009; Carpenter, 2010; Barrows et al., 2014; Maia and Silveira, 2016; Ferrari et al., 2021). Potential sources of related economic gains include reduced crop losses, reduced expenditure on pest control, farmworker safety and health conditions, lower variability of output and consequently, less risk (Sexton and Zilberman, 2012; Smyth et al., 2015; Krishna et al., 2016; Alves et al., 2020). There is also a concern with the non-GM markets regarding the lack of availability of inputs and price differentials (Kalaitzandonakes et al., 2018; Punt and Wesseler, 2018; Oliveira et al., 2020).

Since the mid 1990s, when first-generation GM seeds were commercially introduced, adoption by farmers has grown steadily in industrialized and developing countries as they provide an alternative and more convenient way of controlling weeds and pest damage. By 2018, farmers of 26 countries have cultivated 199.5 million hectares to GM seeds, about 90% of them corresponding to small farmers (ISAAA, 2018). From the first approval of a GM seed in 1996–2018, the number of hectares cultivated with GM grew persistently at 12.8% per year. The main reasons are: 1) the successive approval of GM platform crops (soybean, corn, cotton, and canola) of IR, HT, and IR + HT events in the leading grain producers in the world, notably the US, Canada, Argentina, Brazil, Paraguay, Uruguay, and in the critical grain consumers, India and China; 2) the approval of new traits, highlighting drought resistance sugarcane in Indonesia; 3) the expansion to other countries, like Mexico, Vietnam, and Pakistan - data from ISAAA (2018, p. 7).

From 2006 to 2018, the growth rate is shallow, not significantly different from zero. The main reason is the rapid diffusion of the two main events in the big agricultural countries, reaching the top of 90% of adoption, a huge success. The deceleration is not compensated by the emergence of new countries and new events. Only Portugal and Spain have adopted GM crops in Europe, reflecting the persistence of bans (Oliveira et al., 2020). The heterogeneity of the diffusion processes has been firmly determined by the gains from adoption in the leading agricultural exporters in the world in comparison with other agricultural countries, according to Brookes and Barfoot (2018, 2020).

On the environmental front, benefits related to adoption of GM seeds have been argued based on findings about pesticide use and agricultural practices (Klümper and Qaim, 2014; Datta et al., 2019; Kranthi and Stone, 2020). Insect resistant (IR) cotton has been found to reduce the use of insecticides and therefore to produce environmental, health and safety gains (Huang et al., 2002; Qaim and Zilberman, 2003; Qaim and Janvry, 2005; Qiao, 2015; Veettil et al., 2017). Tabashnik and Carrière (2017) analyze the global monitoring data reported during the first 2 decades of transgenic crops and identified the increase of pest resistance to Bt proteins (Cry and Vip)^1^. They suggested adopting agricultural practices to lessen the adverse effects of pest resistance.

Herbicide tolerant (HT) soybeans have been found to change the mix of herbicides applied towards less toxic ones and to allow the use of no-till cultivation techniques, leading researchers to conclude (tentatively) that they also produce environmental benefits (Fernandez-Cornejo et al., 2002; Qaim and Traxler, 2005; Brookes and Barfoot, 2018; Kalaitzandonakes et al., 2018). However, the diffusion of herbicide-tolerant events associated with a minimal variety of herbicides has generated herbicide resistance with a potential of compromising technology value (Smale et al., 2012; Bonny, 2016; Lamichhane et al., 2017; Schütte et al., 2017).

Although the predominantly favorable evaluation of impacts, a report of the US National Academies of Sciences Engineering and Medicine casts doubts on the productivity and environmental gains that were promised when GM seeds were first introduced (National Academies of Sciences, 2016). Based on a thorough review of evidence accumulated over the last two decades, the report concluded that IR traits in cotton and maize crops decreased the gap between potential and actual yields when targeted pests were a significant source of losses even with chemical control. Nevertheless, when examining data on overall yield per hectare for cotton, maize and soybeans reported by the US Department of Agriculture, the report found no evidence that GM traits have substantially increased the rate at which the US is increasing agricultural yields (National Academies of Sciences, 2016).

Regarding pesticides use, the report found that IR traits have decreased the number of insecticide applications and of kilograms of active ingredients per hectare applied on maize and cotton crops. For HT traits, on the other hand, the evidence on the amount of herbicide per hectare of crop is mixed, with studies that found initial decreases in total amount in soybean crops that were not sustained over time, mostly due to increased resistance of weeds to herbicides (National Academies of Sciences, 2016). The report also warns that analysis that find overall increase or decrease in kilograms of herbicides per hectare can be misleading, since some herbicides are effective at much lower rates than others and changes in applications rates per hectare do not consider changes in the quality mix of herbicides applied.

Recently, some studies raised concerns about the soybean system of weed control, challenging the idea of the social and environmental benefits of the usage of glyphosate in GM crops (Dias et al., 2019). After 25 years following the initial GM diffusion, environmental concerns, and some critics of the performance of GM cultivars, are still in place, even in countries like Brazil and Argentina, which are highly competitive in soybean, corn, and cotton. Brazil is ranked second in GM adoption, justifying the importance of investigating the environmental impacts.

The paper proceeds as follows. The second section evaluates the environmental impact of the use of GM plants that are herbicide tolerant and insect resistant. Field research covers the 2009–2013 period with a particular feature regarding the rapid diffusion of GM varieties: the seed supply was predominantly of non-stacked GM seeds, and many growers use conventional types. One relevant section’s contribution is to compare each GM type on the market with the environmental impact of the use of conventional seeds. The section innovates relative to previous works by employing a broader measure of environmental impact that considers toxicity levels and risk of exposure in evaluating the effects of pesticides for different dimensions of the agricultural system. It allows for uncovering environmental impacts that have been hidden by the qualitative nature of the change in the mix of pesticides used.

The third section provides an analysis of the environmental effects related to the use of pesticides arising from the adoption of IR cotton and HT soybean seeds. The fourth section discuss the quick diffusion of stacked GM that partially contributes to reducing the criticism of the environmental impacts, combining the reduction of insecticide usage with the crop management only possible with HT traits to map the new trends in genetic modified crops, from stacked genes in soybean to novelties based on gene editing. The existence of technological variety for soy and mainly in corn confirms the relevance of GM traits to Brazilian agriculture. The second part of the section discusses the future contributions of plant breeding technologies with attention to climate change effects. The fifth section summarizes the contribution of the paper and its main conclusions.

## 2 Evaluation of environment impact of GM seeds in brazilian agriculture

### 2.1 Methodology

#### 2.1.1 Formulating the evaluation hypothesis

The environmental impact related to pesticides use of GM seeds in Brazil demands a careful assessment, not presented yet in the literature. In the period 2009 up to 2013, as pointed above, the diffusion of stacked GM seeds was going fast, but it was possible, in this period, to find growers choosing HT or IR, allowing the field research to treat HT and IR traits separately.

Soybean seeds engineered with HT traits are the result of the transfer of part of the genetic code of a soil bacterium, *Agrobacterium tumefaciens*, which allow the plant to metabolize the herbicide glyphosate. In 1998, soybean varieties tolerant to the herbicide glufosinate were introduced under the commercial name Liberty Link. Those herbicides target a large variety of broad-leaf and grass weeds species but cause severe damages to conventional crops when applied after germination (post-emergent weed control). The primary reason given for the rapid diffusion rate of those seeds, notably the Roundup Ready ones, is the simplicity of the glyphosate-based weed control, which allows farmers to concentrate on one herbicide to control a wide range of weeds. In addition, it also proved more convenient for farmers since the timing of application can be extended beyond soybean flowering and the maximum size of weeds that are effectively controlled is greater compared with other postemergence herbicides (Carpenter & Gianessi, 1999). Herbicide related cost savings have also been pointed as one of the reasons for adoption, since glyphosate patent expired in the year of 2000, allowing the entry of new suppliers, and lowering the price of glyphosate-based herbicides (Qaim, 2009). Hence, from the point of view of farmers, HT soybeans have been shown to be both technically and economically advantageous, which explains the rapid diffusion they have displayed.

This description of the effects of the HT trait on the plant allows us to formulate two working hypotheses on how it changes the amount of herbicides that farmers choose and the corresponding environmental effect. First, since the HT trait makes the plant tolerant to some specific herbicides—the ones with active ingredients that the plant is now able to metabolize—it can be seen as a technical complement to those chemicals. Hence, we expect the HT trait to induce farmers to use more of the herbicides that the plant is tolerant per hectare. As for the environmental effect, since farmers use more of less toxic chemicals, this should be weighted against the way they substitute away from other more toxic herbicides. If this substitution is strong enough, it is possible that the net effect is a reduction on the environmental impact in terms of general toxicity of the weed-control strategy. On the other hand, if this substitution is weak, the net effect would be an increase in the general toxicity of the weed-control strategy since the additional low-toxicity herbicide would be used on top of high-toxicity ones. We summarize these hypotheses as the following:

1) HT trait increases the amount per hectare of some herbicides applied to the crop. Specifically, it increases the amount of herbicides that the plant becomes tolerant to.
2) Since the herbicides that the plant becomes tolerant to are of lower toxicity, the net environmental effect depends on the strength of substitution among herbicides of different toxicity levels.

IR seeds are engineered to produce a natural toxin found in the soil bacterium Bacillus thuringiensis (Bt), which is lethal to a number of caterpillars (rootworms, earworm, bollworms) pests but not to mammals^2^. IR crops have also been considered technically and economically efficient for producers. The most straightforward reason is related to savings in insecticides applications (which spans savings in labor time, machinery use, aerial spraying etc.) targeted to bollworm killing. Specifically, in regions with high insect infestation, typical less developed countries in tropical weather regions, and high rates of insecticide use, the potential for reduction is conversely high (Qaim and Zilberman, 2003; Kathage and Qaim, 2012; ISAAA, 2018).

IR seeds have also been found to increase yields relative to non-GM ones since the toxin produced by the plant, compounded with the insecticides usage, reduces losses due to insect attacks (Qaim, 2009; Veettil et al., 2017). In fact, it has been argued that yield and insecticide reduction effects are closely related: farmers facing high pest pressure and still using low rates of insecticides. Besides, it has also been considered a more efficient tool for managing the risk of pest attack than reactive application of insecticides (Crost and Shankar, 2008) which has been translated in reduced crop insurance premium. Other benefits relate to improved farm workers' safety conditions and shorter growing seasons (Brookes and Barfoot, 2018, 2020).

As for the HT trait, we can formulate two working hypotheses for the effects of the IR trait on the amount of insecticides used and the related environmental impact of the insect-control strategy. Since the plant produces a natural toxin that substitutes insecticides aimed at some types of bollworms, the IR trait works as a substitute for chemical insecticides and hence reduces the amount that farmers have to apply. The environmental effect should be straightforward: fewer chemicals applied to the plant should lead to a less toxic pest control strategy. We summarize these two hypotheses in the following statements:

1) The IR trait reduces the amount of insecticides that farmers apply to the crop.
2) The IR trait reduces the environmental damage related to the application of chemical insecticides.

This discussion suggests that measuring environmental impacts associated with pesticide use is not straightforward. For HT traits, specifically, the net effect on environmental impact is an open issue. Economists that studied it have focused on the change in the mix of herbicides to conclude that there are environmental gains allowed by the use of HT traits. Nevertheless, we argue that weak substitution might undermine this conclusion as we show in the analysis that follows on the next sub-sections.

#### 2.1.2 Empirical strategy

In the empirical analysis, we use a unique farm-level dataset originated from a survey conducted by a Céleres Consultancy, in Brazil. The survey collected data on production, revenue, costs, biotechnology adoption and pesticides used. Information on pesticide use was collected for harvest seasons 2009–2013 and covers 1,030 farms.

The dataset is disaggregated by fields, within a farm, cultivated with conventional or GM seeds. In other words, for each farm, we have potentially multiple observations related to fields cultivated with conventional or GM seeds. This setup allows us to explore within-farm variation between fields cultivated with conventional and GM seeds to identify the effect of biotechnology traits on the use of pesticides and corresponding environmental impact. This identification strategy holds constant all farm-level characteristics that might affect simultaneously the choices of pesticide use and biotechnology adoption such as: management skills, input/output prices, location, weather shocks, etc. Hence, for instance, if soybean farmers that adopt biotechnology have some intrinsic preference for pest management strategies that are more intensive in herbicides than mechanical control (like tillage) the effect of GM traits could be overestimated. Likewise, if cotton farmers that adopt IR traits are more efficient and also use less insecticide in their pest management strategies, the effect of IR trait will be underestimated^3^. The use of within farm variation, i.e., comparing the pesticide use and corresponding environmental impact for farmers that cultivate fields with conventional and GM seeds, gets around these sources of bias on the coefficient that measures the effect of the GM trait.

The farms surveyed represent large operations with potentially large environmental impacts associated with the scale of production and pesticides use. For cotton growers, the average total planted area is 1,888.48 ha, ranging from 50 ha to 26,774 ha. For soybean growers, the average total planted area is 857.88 ha ranging from 5 ha to 11,000 ha. In terms of experience, farmers report an average of 26.95 and 33.33 years for cotton and soybeans respectively. This indicates they have a high level of working experience in the activity.

We measure the environment impact as two outcome variables: quantity (Kg/ha) of active ingredients of chemicals and the Environmental Impact Quotient (EIQ) index (Kovach et al., 1992). This measure of environmental impact of pesticides was designed to capture risks associated with both toxicity levels and exposure to chemical pesticides on three components of agricultural systems: farm worker, consumer and ecological. Hence, the EIQ index provides a more complete picture than just the composition of the mix of pesticides used, or the analysis of kilograms of active ingredients applied to crops, allowing for an adequate weighting of pesticides of different toxicity levels (National Academies of Sciences, 2016).

The use of the EIQ index represents a considerable advancement over previous studies that relied on an increased share of less toxic chemicals in the total quantity (Kg/ha) of herbicides applied in HT soybeans fields since this measure cannot capture environmental effects due to substitution between herbicides. Concretely, if the increase in the use of less toxic herbicides is not accompanied by a sufficient decrease in more toxic ones, the new mix of herbicides induced by HT seeds can be more harmful than the one induced by conventional seeds. The EIQ index calculated for field operations allows us to adequately weight pesticides of different toxicity levels and gets around the difficulties of looking only at the quantity mix of pesticides used.

We estimate linear regression models for cotton and soybean crops separately. The dependent variables are quantity (kg/ha) of pesticides used (insecticides for cotton and herbicides for soybean) and EIQ index for each field. The traits considered are the most common ones for each crop: IR for cotton and HT for soybean. The estimated equations have the following form:

$$y_{\mathrm{itf}}=\alpha +\mathrm{\beta trai}t_{f}+\gamma_{i}+\theta_{t}+\varepsilon_{\mathrm{itf}}\;$$

Subscripts *i, t* and *f* indicate farmer, year and field (each field cultivated with conventional or GM seed). We include farmers (*γ*_*i*_) and time dummies (*θ*_*t*_) that capture farm-specific and year specific effects. Although these variables are orthogonal to the field level effects that we are interested, therefore not affecting the point estimates, they provide efficiency gains in the estimation (lower standard errors) that prove worth keeping them.

### 2.2 Results

#### 2.2.1 IR traits in cotton

Table 1 shows estimates of the effect of adoption of IR trait in cotton crops for quantities (Kg/ha) of active ingredients of insecticides and total pesticides applied (models 1 and 2) and for the field EIQ for insecticides and total pesticides (models 3 and 4). Estimates are for the sample of farmers that use both conventional and IR seeds^4^.

**TABLE 1:** Effect of IR trait on quantity and field EIQ of insecticides and total pesticides.

	(1)	(2)	(3)	(4)
	Insecticides	Total	Insecticides	Total
IR trait	−1.025^***^	−1.005^***^	−31.449^***^	−33.237^***^
	[0.185]	[0.242]	[5.596]	[6.844]
			
Constant	1.168	3.418^**^	50.686	111.582^**^
	[1.146]	[1.131]	[34.214]	[34.097]
N	186	186	186	186
*r* ^2^	0.822	0.861	0.848	0.873
Mean of Dep. Var. ^+^	4.67	11.01	154.94	304.66

Models (1) and (2): kg/ha of active ingredients of insecticides and total pesticides.

Models (3) and (4): field EIQ, for insecticides and total pesticides.

Restricted sample: farmers that use conventional and IR, seeds.

Robust standard errors in brackets.

* *p* < 0.05, **p < 0.01, ***p < 0.001.

+ Conventional seeds.

The coefficient of the IR trait indicates that it allows a reduction of 1.025 kg/ha of active ingredients of insecticides applied to cotton fields. For total pesticides the point estimate is a bit lower in magnitude (−1.005) but not statistically different from the coefficient on insecticides. This indicates that reduction in active ingredients comes mostly from insecticides. When comparing this reduction with the average of 4.67 kg/ha of active ingredients of insecticides applied in fields cultivated with conventional seeds, the reduction amounts to approximately 22% of active ingredients. Relative to total pesticides, the proportional reduction amounts to 9%^5^.

Consistent with the reduction in quantity of insecticides, the coefficient on EIQ indicates a reduction of 31.49 EIQ points for insecticides and 33.237 for total pesticides. To gain some perspective on this magnitude, in comparison with the general classification of active ingredients for insecticides, this is higher than the median EIQ index of 32.07. When compared to the average of 154.94 EIQ points for insecticides in fields cultivated with conventional seeds, this amounts to a reduction of 20%^6^. Hence, it can be considered a significant reduction in terms of environmental index.

Those results are consistent with the current state of the literature on environmental effects of IR seeds. Studying IR cotton seeds in India, Qaim and Zilberman (2003) found reduction of 1 kg/ha on average use of insecticides (70% compared with the baseline conventional field) while Qaim and Janvry (2005) found reductions between 1.2 kg/ha and 2.6 kg/ha of active ingredients used in Argentina, which represents about 50% reduction in comparison with conventional plots. For China, Huang et al. (2002) found even bigger reductions of about 49 kg/ha of average insecticide use (80.5% compared to the average of 60.7 kg/ha in conventional fields).

#### 2.2.2 HT trait in soybean crops

Table 2 shows estimates of the effect of adoption of HT trait in soybean crops for quantities (Kg/ha) of active ingredients of herbicides and total herbicides applied (models 1 and 2) and for the field EIQ for herbicides and total pesticides (models 3 and 4). Estimates are for the restricted sample of farmers that use both conventional and IR seeds.

**TABLE 2:** Effect of HT trait on quantity and field EIQ of herbicides and total pesticides.

	(1)	(2)	(3)	(4)
	Herbicides	Total	Herbicides	Total
HT Trait	0.983^***^	0.979^***^	13.685^***^	14.013^***^
	[0.084]	[0.091]	[1.545]	[1.941]
			
Constant	1.315^***^	4.756^***^	24.241^***^	85.928^***^
	[0.042]	[0.061]	[0.980]	[1.225]
N	182	182	182es	182
r2	0.837	0.904	0.839	0.939
Mean of Dep. Var^+^	1.76	3.28	30.76	82.35

Models (1) and (2): kg/ha of active ingredients of herbicides and total pesticides.

Models (3) and (4): field EIQ, for herbicides and total pesticides.

Restricted sample: farms that use both conventional and HT, seeds.

Robust standard errors in brackets.

* *p* < 0.05, **p < 0.01, ***p < 0.001.

+ Conventional seeds.

The estimates show that adoption of HT trait increases the quantities (Kg/ha) of active ingredients of herbicides used by 0.983 kg/ha. For total pesticides, the coefficient is slightly smaller, indicating that the increase comes mostly from herbicides. When comparing this increase with the average of 1.76 kg/ha of active ingredients of herbicides applied in fields cultivated with conventional seeds, the increase amounts to approximately 55.8% of active ingredients. Relative to total pesticides, the proportional increase amounts to 30%.

The coefficients for the EIQ index shows that adoption of HT seeds increases the environmental impact of herbicides by 13.685 points. This represents a proportional increase of 44.4% relative to fields cultivated with conventional seeds^7^. For total pesticides the increase in the EIQ index is slightly bigger. In comparison with the general EIQ classification for herbicides, this is lower than the median value for EIQ index of 19.5. The EIQ for glyphosate is also larger than this result: 15.33. In the sample, the mean EIQ for herbicides is 37.6 and for all pesticides 89.36.

Table 3 breaks the effects on herbicides by categories of toxicity level (1–4 in decreasing order). Categories three and four show significant increases of 0.597 and 0.465 kg/ha of active ingredients respectively while categories one and two show reductions of 0.083 and 0.008 kg/ha (not statistically significant) respectively. Hence, the increase in less toxic herbicides is almost elevenfold the reduction in more toxic herbicides. This result shows that substitution among herbicides of different toxicity classes is very low, which indicates that this channel of environmental benefits is very limited. In other words, farmers adopting HT seeds are increasing the use less toxic herbicides on top of the more toxic ones. Besides weak substitution, this result also supports the idea that weed infestation is not systematically correlated with the adoption of HT seeds, which reinforces our confidence that the bias in the point estimates due to this channel might be very weak.

**TABLE 3:** OLS estimates of effects of HT trait on quantity (Kg/ha) of herbicides per toxicity level.

	(1)	(2)	(3)	(4)
	Herbicides 1	Herbicides 2	Herbicides 3	Herbicides 4
HT Trait	−0.083^***^	−0.008	0.597^***^	0.465^***^
	[0.020]	[0.051]	[0.095]	[0.087]
Constant	0.041	0.046	−0.154	1.388^***^
	[0.042]	[0.045]	[0.304]	[0.307]
N	180	180	180	180
*r* ^2^	0.887	0.788	0.851	0.844
Mean of Dep. Var^b^	0.23	0.22	0.78	0.51

Restricted sample: farms that use both conventional and HT, seeds.

Robust standard errors in brackets.

* *p* < 0.05, **p < 0.01, ***p < 0.001.

+ Conventional seeds.

Toxicity levels 1–4 in decreasing order (from more to less toxic). Herbicides based on Glyphosate are considered of lower toxicity level. Increases in less toxic herbicides (levels 3 and 4) are about *elevenfold* the decreases in more toxic ones (levels 1 and 2).

## 3 Discussion

This section provides an analysis of the environmental effects related to the use of pesticides arising from the adoption of IR cotton and HT soybean seeds. Using within-farm variation across fields treated with conventional and GM seeds, the results have shown that IR cotton reduces the number of insecticides applied to cotton crops. On the other hand, HT soybean leads to more use of herbicides. Analysis using the EIQ index shows that IR cotton reduces the environmental impact by about 20% in the treated fields compared to fields cultivated with conventional seeds. This is consistent with the previous result on Kg/ha of insecticides and confirms the environmental impact saving nature of the IR technology. The resulting environmental effects for HT soybean, on the other hand, are found to be negative. The estimates imply an increase of 36.1% on the impact of herbicides compared to fields cultivated with conventional seeds.

Regarding the quantities of herbicides of different toxicity levels, the results showed an increase in the use of lower toxicity herbicides and slight reductions for higher toxicity ones. This finding indicates very weak substitution among herbicides, which explains the higher environmental impact associated with these chemicals caused by adoption of HT soybeans.

It is worth it summing up the contributions of empirical analysis in three points. First, it contributes to uncovering environmental effects that have been hidden by the qualitative nature of the mix of herbicides induced by the HT trait. Second, ecological policymakers designing policies for biotechnology adoption might consider this new evidence to differentiate among GM traits that produce positive or negative externalities. Finally, as the composition of the EIQ index suggests, the environmental impact of pesticides can have multiple dimensions that might involve farmworker health and safety, consumer safety, and ecological effects. Hence, the results on HT soybeans point to additional avenues of work that should be taken to evaluate each of these possible channels since they can also affect other vital outcomes.

The results also suggest that previous findings on the environmental effects of HT soybeans might have been biased by the qualitative nature of the mix of herbicides^8^. Fernandez-Cornejo et al. (2002) found evidence of reduction in the use of acetamide herbicides and increase in the use of glyphosate in United States. Qaim and Traxler (2005) studying HT seeds in Argentina found a total increase of 107% in the use of herbicides, which are divided in a decreases of 87% and 100% in toxicity classes two and three, respectively, and an increase of 248% in toxicity class four. The authors suggest that this change is basically due to the use of no-till farming by adopters of HT soybeans.

Our results are not incompatible with those previous findings. In fact, we also observe a change in the composition of the mix of herbicides used towards less toxic products. This movement is predicted by the theoretical analysis that shows how the HT trait increases the value of marginal product of herbicide (glyphosate) and, therefore, the optimal amount used. On the other hand, we also find very weak substitution among herbicides of different toxicity classes, which suggests that the environmental impact of herbicides in being magnified. The analysis with the EIQ index confirms that this is not only a possibility: even inducing more use of a less toxic herbicide, HT seeds cause higher environmental impact, even when controlling for the use of no-till farming.

## 4 The economic and environmental benefits of stacked GMOs and the opportunities generated by scientific advances in plant breeding

### 4.1 Stacked varieties have diffused quickly

A novelty characteristic of the last decade is the emergence of stacked genes. In 2011, stacked GMOs were cultivated in 42.2 million hectares –23.4% of the global area covered by transgenic seeds. Since then, plantations of this kind of cultivar registered strong growth, reaching 80.5 million hectares in 2018, representing 42% of the global area dedicated to GM crops.

Table 4 presents some insights that sum up the adoption situation of three main GM seeds in 2011 and 2018. These two dates are related to the studies we show in the paper: the first year is precisely in the middle of the 4-year sample to evaluate the environmental impact of IR in cotton and HT in soybean. The figures for the second year, 2018, call attention to the rapid diffusion of stacked genes that solves some caveats^9^ generated by the need for growers to choose between HT and IR traits.

**TABLE 4:** Some insights of the Adoption of GM Seed in Brazil, 2011 and 2018.

2011 Crop season									
Crop	Total area (ha)	Adoption Rate (as % of Total Area including GMO Crops + Non-GMO Crops)				Area with GM Traits (Millions of Hectares)			
		IR	HT	IR/HT	Total	IR	HT	IR/HT	Total GMO
Soybean	25.0	0.0%	82.4%	0.3%	82.7%	0.0	20.6	0.07	20.7
Maize (summer + winter)	14.04	30.6%	7.5%	26.9%	65.0%	4.3	1.05	3.8	9.1
Cotton	1.55	8.5%	14.3%	16.2%	39.0%	0.132	0.222	0.251	0.605
Total Soybean + Maize + Cotton	40.6	10.9%	53.9%	10.1%	74.9%	4.4	21.9	4.1	30.4
**2018 Crop Season**									

**Crop**	**Total Area (ha)**	**Adoption rate (as % of total area including GMO crops + Non-GMO crops)**				**Area with GM traits (millions of hectares)**			
		**IR**	**HT**	**IR/HT**	**Total**	**IR**	**HT**	**IR/HT**	**Total GMO**
Soybean	36.39	0%	40.1%	55.5%	95.6%	0.0	14.6	20.2	34.8
Maize (summer + winter)	17.3	25.4%	3.7%	59.5%	88.7%	4.4	0.646	10.3	15.3
Cotton	1.2	8.2%	14.4%	62.8%	85.4%	0.098	0.173	0.754	1.025
Total Soybean + Maize + Cotton	54.9	8.2%	28.1%	56.9%	93.2%	4.5	15.4	31.3	51.2

Source: James (2011) and ISAAA (2018).

Inspecting Table 4, soybean, motivated by the rise in prices, contributed to pushing the GM total area. Call attention to the preference of growers for HT, performing 82.4% of the total area of soybean, with 20.6 million hectares. Despite the late approval of IR traits to corn in Brazil (in 2007), these traits performed 57.5% of the corn area in 2011. Cotton is in the last position, even with the importance of controlling bollworms.

The situation has changed sharply in 7 years. During the decade following the 2011 crop, research and development efforts in Brazil prioritized crosses between different lineages of first-generation GMOs to generate breeds able to express both the HT and IR biotech traits coming from their genitors. Stacked GMOs can be classified into four different types: 1) genes that confer resistance to multiple insect species; 2) the expression of the Bt insecticidal protein in parallel with tolerance to glyphosate herbicide; 3) genetic sequences ensuring a simultaneous tolerance to different types of herbicide; 4) other types of biotech traits capable of enhancing plant tolerance to droughts and/or improving its nutritional content (Pellegrino et al., 2018).

In the Brazilian case, the great leap in the adoption of stacked cultivars started in the 2013/2014 crop with the release of Monsanto’s Soja Intacta™, which expresses simultaneously both biotech traits, HT and IR. In a mere 5 years, Intacta™ ‘s cultivation area went from 2.3 million hectares in 2013 to 20.2 million hectares in 2018, making this cultivar the GMO with the largest diffusion during the 2010s (ISAAA, 2018). In the face of the increasing replacement of soybean varieties which express only tolerance to glyphosate by Intacta™, the HT + IR seeds became predominant in the Brazilian soy culture. Moreover, Table 4 reveals that Brazilian GM maize and cotton crops experienced a similar situation, increasingly favoring stacked GMOs with respect to the first generation ones.

The revealed preference for stacked genes calls attention to the importance of integrating the modules that compose the grain production. It means that from soil preparation to harvesting, the combination of GM traits facilitates crop management and reduces risks associated with critical delays in the sowing period (Carauta et al., 2017). The use of stacked genes forcibly reduces the GGE emissions by eliminating some tasks in soil preparation, sowing, and pest control and provides a kind of insurance to growers once the plant is resistant to essential pests (Alves et al., 2020).

In the section dedicated to evaluating the environmental impact of GM seeds, we use a unique farm-level dataset documenting the adoption of GM seeds between 2009 and 2013 by commercial farms in Brazil. Table 4 suggests that data of the soybean, maize and cotton plantations Environmental Impact Index encompass a period characterized by an ample predominance of first generation GMO cultivation. Since then, the adoption of stacked GMOs has registered a strong growth, reaching 31.1 million hectares in 2018 (60.94% of the of the Brazilian crop area dedicated to transgenic seeds).

The fast pace of diffusion of stacked GMOs in Brazil and worldwide^10^ has motivated a variety of studies about the economic and environmental impact of this technological innovation. These works point to the gain in agricultural productivity, the farmers’ increasing profits, the decrease in the use of crop protection chemicals, and the reduction of carbon emissions, as the main benefits of stacked seeds compared with single-trait biotech GMOs (Waquil et al., 2013; Pellegrino et al., 2018; Brookes and Barfoot, 2020).

In a meta-analysis published in Nature Scientific Reports, Pellegrino et al. (2018) reviewed 76 scientific publications in order to analyze the economic impact of four types of GM maize seeds^11^. The authors determined that the decrease in pesticide application and the increase in crop yield were more significant in the areas planted with quadruple stacked hybrids. The authors have found that the stacking of genes has been successful in widening full protection against pests and delaying the appearance of insects resistant to the applications of agricultural biotechnology.

Studies comparing HT + IR soybean seeds with single-trait biotech cultivars of the same grain obtained similar results to the ones found in the case of maize. According to Brookes and Barfoot (2020), the adoption of Soja Intacta™ provided to South American growers economic benefits equivalent to 10.2 billion dollar during 2013–2018. This implies that for every US$ 1 invested in Intacta™ technology, the growers received approximately US$ 3.88 of additional profit. This economic gain was the result of a production increase of 27.3 million tons of soybean (considering the productivity increases obtained from a total cultivated area of 73.6 million hectares during 6 years) and the expense reduction in weed and pest control.

Intacta ™ soybean cultivation reduced chemical protection application in such magnitude as to imply a fall in the Environmental Impact Quotient (EIQ) of GM soybean crops:

“Intacta soybeans have enabled soybean growers to reduce the average number of insecticide treatments by about 4 (from an average of 8–10 sprays on conventional or GM HT only crops) in Brazil […] Based on these savings, in 2018, the use of this technology resulted in a reduction of four million kg of insecticide active ingredient use, equal to 13.1% of total insecticide used on the soybean crops in the four countries. The EIQ saving in 2018 was equal to −13.8%. Over the 6 years, the total insecticide active ingredient usage saving has been 14.9 million kg (−8.2%) and the associated environmental impact, as measured by the EIQ indicator fell by 8.6% (Brookes and Barfoot, 2020, p.98-99).”

The authors also highlighted that the adoption of Soja Intacta ™ has reduced the level of greenhouse gas emissions associated with soybean cultivation. This is mainly due to fuel savings caused by the reduction by half of aerial spraying in areas planted with HT seeds or traditional varieties. Furthermore, by eliminating unwanted and competing plants, Intacta™ technology facilitates the transition from traditional planting systems (predominant in non-transgenic seed cultivation) to direct planting systems, far less dependent on soil preparation operations, such as mechanized plowing (Brookes and Barfoot, 2018). For these reasons, after 5 years of its adoption, Intacta ™ technology contributed to a carbon dioxide emission reduction equivalent to the removal of 3.3 million cars from the roads (ISAAA, 2018).

### 4.2 Limitations of gene stacking techniques and future implications of the new genome editing technologies

Despite the advantages provided by stacked GMOs for pest control and reduction of greenhouse gas emissions, some researchers warn about the difficulties the seed industry has faced to adapt itself to climate change, specially, in abiotic stress situations. Graff et al. (2009) raised the hypothesis that the pace of development of seeds that need less water for growing fell short of what would be expected. Throughout the decade following this study, the diffusion of biotech traits capable of increasing drought tolerance in plants has also been slow^12^.

A climatic event in Argentina elucidated one of the main challenges the seed industry will face in the following decades. During the 2018/2019 crop season, “a severe drought during the peak summer months reduced the area planted to biotech soybean” (ISAAA, 2018, p. 18–19), which led to a reduction of production and put in evidence a considerable limitation, inherent to the cross-hybridization techniques used in the development of stacked GMOs.

The stacking of many biotech traits tends to compromise the myriad of other agronomic attributes not controlled by the transferred genes, which can ultimately reduce the physiological quality and productivity of the host plant. If on one hand, the technical limitations of transgenic processes and gene stacking has hindered the diffusion of new agronomic traits (Qaim, 2020), on the other, various authors are hopeful that new genome editing techniques based on CRISPR-Cas9 can, in the future, alleviate the above technological obstacles (Vats et al., 2019; Zaidi et al., 2019).

Genome editing techniques are already being used to develop tolerance to abiotic stress in soybean, maize, rice, wheat, and bean cultivars, as well as in several other cultivations. Therefore, there exists an expectation that the CRISPR-Cas9 system revolutionizes the development process of agronomic traits, enabling the expression of a much larger number of traits than the ones currently observed in the GMOs existent in the market (Vats et al., 2019; Zaidi et al., 2019; IHS Markit, 2020; Qaim, 2020)^13^.

Even though gene-edited seeds are still not used at a commercial scale and, up to this moment, their economic and environmental impact cannot be observed nor quantified (Qaim, 2020), it is already possible to point out the main technology holders of the most important editing technologies as well as to indicate some cultivars already approved by North American and Brazilian regulatory authorities.

According to Egelie et al. (2016, p.1028), the Boston academic cluster (consisting of the Broad Institute, MIT and Harvard University) and the University of California, Berkeley jointly concentrate proprietary control of the main components of the CRISPR-Cas9 system. The cluster was responsible for 20% of the patents filed in this field up to 2016 (131 documents). The University of California owned a smaller portfolio, with 14 patent families which, however, included some of the essential enabling technologies for the whole system. In this way, such institutions held full control of the medical applications of the CRISPR-Cas9 technology. On the other hand, the control of agricultural and food applications of the same technology was distributed in a more balanced way in the corporate sector, with Dow-DuPont playing a prominent role.

The work of Egelie et al. (2016) is crucial to understand the uncertainty involving the CRISPR system at that moment. The first great uncertainty involved the property of the Cas9 molecular scissors. The Boston cluster and the University of California, Berkeley filed, almost simultaneously, patents claiming the discovery. The USPTO granted the ownership of the enzyme to the Boston cluster. Soon after, the University of Berkeley filed a request for patent revocation to the same agency. In spite of this conflict, both academic groups created their own startups. Caribou Biosciences is a commercial spin-off of the University of California, Berkeley, in the same way that Editas Medicine was created by the MIT/Broad Institute.

In both cases, the startups were granted exclusive patent licenses for commercializing the biotechnologies developed by the original universities, so companies that decide to use the Cas9 tool would inevitably have to negotiate sublicenses with one or both of the above startups. Some statements from Caribou and Editas led Egelie et al. (2016) to fear that both startups would opt for the internal development of new products instead of transferring the technologies to the seed industry, which could ultimately create legal obstacles for the development of new agricultural applications of CRIPSR-Cas9.

Fortunately, this pessimistic scenario did not materialize. As Zhang et al. (2019) highlighted, the scientific community identified other molecular scissors (*e.g.* Cas12a, Cas13a, CasX, etc.) capable of replacing Cas9 in the CRISPR system. Furthermore, the lead companies in the seed industry did not have much difficulty negotiating technological licenses with commercial representatives of both academic groups disputing the ownership of the Cas9 enzyme (IHS Markit, 2020).

For instance, Dow-DuPont was one of the first companies to negotiate a technological sublicense for the purpose of exploring Caribou Biosciences agricultural technologies (Egelie et al., 2016), which later was inherited by Corteva (a spin-off from this conglomerate which become a standalone company). More recently, Corteva negotiated a number of tripartite agreements of intellectual property which involved, at the same time, the academic institutions composing the Boston cluster and several bioinformatic companies, such as the J. R. Simplot Company, Yield10 Bioscience and Amfora (IHS Markit, p.2020). These tripartite agreements established legal conditions for the utilization of the molecular scissors developed by MIT and Harvard to do genome-editing of Corteva’s cultivars.

By virtue of these joint research efforts, Corteva obtained its first gene-edited cultivar, namely, the waxy corn hybrids (hybrid corn with waxy starch). In short, Corteva’s scientists disabled the amylose gene with the intention of raising the level of amylopectin in corn starch, thus benefiting the frozen food, dye and glue sectors. The waxy corn hybrids received the approval of the United States Department of Agriculture (USDA) in 18 April 2016 (see Chart 1), going down in history as the second cultivar developed from the CRISPR system to be released for planting and commercialized in the United States. Since then, approval events have only multiplied in that country.

**CHART 1:** Gene-edited cultivars approved by the United States Department of Agriculture (USDA).

Approval date	Crop	Agronomic trait
04/13/2016	Mushroom	Do not turn black on the cut
04/18/2016	Maize	Increase in Amylopectin levels
11/15/2016	Potato	Do not turn black on the cut
12/02/2016	Potato	Do not turn black on the cut
08/29/2017	False flax	Increase in Omega-3 levels
10/16/2017	Soybean	Salt and drought resistance
11/25/2017	Alfalfa	Enhancement of digestibility
01/12/2018	Maize	Fungal resistance
03/19/2018	Maize	Productivity enhancement
03/20/2018	Wheat	Higher fiber content
05/14/2018	Tomato	Improvement of the harvesting process
08/06/2018	Pennycress	Improvement of oil quality
11/07/2018	False flax	Increase in Omega-3 levels

Source: USDA, adapted from (Venâncio, 2019, p.31).

The approval timetable transcribed in Chart 1 has influenced the Brazilian regulatory authorities. On 15 January 2018, the National Biosecurity Technical Committee (CTNBio) from Brazil enacted the Normative Resolution n°16 (RN16) establishing regulatory parameters for the gene-editing technologies. The RN16 resolutions follow the USDA positioning, namely that the requests should be analyzed on a case-by-case basis according to the method of production of the cultivar. It follows that the existence or not of DNA sequences coming from other species represents the main criterion to differentiate the GMOs from gene-edited cultivars. In the absence of exogenous DNA fragments and/or other applications of recombinant DNA technology, the varieties developed through the CRISPR system should be considered as non-transgenic conventional organisms (Eriksson et al., 2019).

In view of the alignment between the RN16 and the North American regulatory framework, the request for regulation of the waxy corn hybrids in Brazil made by Corteva happened quickly. In a polling that took place in November 2018, the CTNBio granted to waxy corn hybrids the condition of conventional organisms, becoming one of the first gene-edited cultivars in Brazilian national territory (Eriksson et al., 2019). Very recently, in a CTNBio meeting on 9 December 2021, the Committee approved the first edited sugarcane cultivars in the world. The Cana Flex 1 (enhancement of the digestibility of cell walls) and the Cana Flex 2 (higher levels of sucrose) were developed by the EMBRAPA Agroenergia to facilitate the production of first and second generation ethanol as well as the manufacture of other bioproducts from sugarcane bagasse.

One of the main criticisms aimed at GMOs is related to the concentration of the R&D efforts on just four products with strong commercial appeal–GM maize, soybean, cotton, and canola seeds. Therefore, the vast majority of agricultural crops seem to have become orphan from the productivity gains derived from the application of recombinant DNA technology in agriculture (Graff et al., 2009). Add to this criticism, another one equally relevant, questioning the seed industry focusing on only two biotech traits: HT and IR (Ferrari et al., 2021). When compared with other already released transgenic events, the requests of approval of gene-edited cultivars made in the United States (Chart 1) and in Brazil seem to indicate a much greater balance: 1) between the agricultural crops that could be considered by the new biotechnological advances, and 2) regarding to the range of agronomic traits that might be included in the research and genetic improvement programs.

## 5 Conclusion

GM crops have diffused quickly since 1996, focused on three platform crops and canola, restricted to a few countries, and two main traits: insect resistance and herbicide tolerance. Despite the restrictions, GM varieties were adopted by the more prominent producers and exporters in the world, notably the United States, Brazil, Canada, Argentina, and India (more than 90% of the total GM adopted).

The contrast between “lovers” and “haters” of GM crops has spurred studies to evaluate impacts. Economic gains of GM adoption are not easy to assess once HT varieties are not related to cost reduction; the two main reasons for adopting HT varieties are risk reduction and the simplification of production processes. However, these factors allow the increase in land productivity. Using IR varieties contributes to cost and risk reduction and simplifies the productive processes. Still, it can induce the substitution of pesticides due to the appearance of new and more resistant pests. All these considerations are based on the literature.

Profiting from the unique opportunity to analyze data from a 5-year research field from 2009 to 2013, the paper tests two hypotheses related to IR and HT varieties, using the most paradigmatic crops: cotton in the IR case and soybean in HT. Results from IR are straightforward and adhere to the results verified in the literature: the IR trait reduces the environmental impact by about 20% compared to crops using conventional seeds. There is a reduction in the quantity per hectare of insecticides usage, but more importantly, the GM seeds reduce the impact by using 22% less pesticide. It also contributes to substitute the pesticide usage in 9%, meaning that it is more challenging to replace insecticides in cotton. The substitution effect between pesticides was, in this case, less significant than reduction, so both estimates, quantities, and EIQ have pointed to a positive environmental contribution of GM adoption.

A different scenery was seen in the case of HT adoption in soybeans. In this case, the evaluation based on EIQ indexes has shown to be relevant to answering the research questions proposed. The GM production system used 55.8% and 30% more active ingredients than the conventional system in herbicides and total pesticides. Since glyphosate (the leading herbicide in the GM system) is less toxic than others used in the conventional method, there was room for the substitution effect. The increase in the EIQ index for herbicides is 44.4% and 26.5% of total pesticides, which is quite disappointing. The substitution effect from more toxic (1 and 2 categories) to less harmful was not enough to reduce the environmental damage of the GM system to weed control in soybean in Brazil. The choice of GM seeds has generated managerial advantages and possibilities to intensify land usage (no-till, double cropping) with the side effect that weed infestations are not systematically correlated with adopting HT seeds.

Going beyond the conclusion that the use of the EIQ index is relevant to understanding the environmental impacts of GM in Brazil is the fact that the combination of IR + HT can make GM technology more favorable. The diffusion direction was to adopt stacked GM seeds that avoid a choice between being efficient in weed control and pollutant in controlling pests. Data shows a sharp change in the adoption of GM. In 2011, HT was integrally adopted by whom had GM as a choice. The adoption rate of GM in cotton was low. In 2018, the higher level (more than double) of GM was due to HT + IT traits (63% of the varieties).

Although the diffusion processes have been technology-led, the quick response of the seed industry (the fierce rivalry between innovative firms is still in place) shows the attention to growers' demands and the incentives the technological fees provided to leading firms. The recent investigation of the frontier of plant breeding points to the diversification of traits and cultures, allowing the technology to contribute to problems related to global warming effects in agriculture and overpass the criticism coming from grassroots movements and the people with an urban view of agriculture.

## Data Availability

The raw data supporting the conclusion of this article will be made available under request to the corresponding author.

## Footnotes

1 Insect-resistant genetically modified crops receive genes from *Bacillus thuringiensis*, a Gram-positive bacteria that allows the plant to synthesize proteins that interfere with the absorption of food from insects. The genetic modification uses two types of genes: Cry (parospal crystal) and Vip (vegetative insecticide protein). Combining the different genes of Cry and Vip can amplify the range of insect control by Bt crops.

2 The paper takes bollworm as the primary reference to the various types of caterpillars causing damage to agriculture. “Bt” technology offers farmers resistance in the plants to major pests such as stem and stalk borers, earworms, cutworms and rootworm (*Spodoptera frugiperda, Diatraea spp, Helicoverpa zea* and *Diabrotica spp*) in maize, bollworm/budworm (*Heliothis sp* and *Helicoverpa*) in cotton, caterpillars (*Helicoverpa armigeru*) in soybeans.

3 Céleres Consultancy, from 2009 to 2013, has conducted field investigations in main agricultural areas in Brazil, with a complete range of crop production. The paper only explores the question of the environmental impacts of pesticide usage.

4 The sample is not representative of cotton and soybean agriculture. The “solution” to compare conventional and GM crops results in some bias. However, the empirical strategy allows a correct comparison in terms of the incidence of pests and weeds that are the primary determinant of spraying, favoring the analysis of the effect of the adoption of GM crops.

5 Log-linear specifications shows a decrease of 23% in the amount of insecticides and 8.8% in total quantity of pesticides. We also estimate similar models per toxicity class (I-IV in decreasing level of toxicity) which indicate reductions in all classes, the most prominent effect being for class III (medium-low level of toxicity) with a proportional decrease of 40%. Those results are available upon request.

6 The log-linear specification shows a proportional reduction of 20.1% in the EIQ index for insecticides.

7 Log-linear specifications show a proportional increase of 44.4% in the quantity of active ingredients of herbicides and 26.5% in total pesticides.

8 In fact, the National Academy of Sciences report recommends that “[be]cause of the difference in toxicity in the various chemicals used, researchers should be discouraged from publishing data that simply compare total kilograms of herbicide used per hectare per year because such data can mislead readers.” (National Academies of Sciences, 2016, p. 8, p. 8).

9 The diffusion of stacked genes has two economic effects: a) simplify the decision process of growers related to pest and weed control, reinforcing the feature of GM seeds of reducing productive risk (Alves et al., 2020); b) contribute to the rise of seed prices via royalties (or technological fee), amplifying the menu of technological choices according to the technological level of growers (Foster and Rosenzweig, 2004).

10 In 2011, stacked GMOs were cultivated in 42.2 million hectares, –23.4% of the global area covered by transgenic seeds. Since then, plantations of this kind of cultivar registered a strong growth, reaching 80.5 million hectares in 2018, representing 42% of the global area dedicated to GM crops.

11 The comparison involved the following hybrid corn: i) GM single-trait biotech seeds (lepidoptera resistance); ii) double stack (lepidoptera resistance + glufosinate tolerance); iii) triple stack (lepidoptera resistance + coleoptera resistance + glufosinate tolerance); iv) quadruple stack (lepidoptera resistance + coleoptera resistance + glufosinate/glyphosate tolerance).

12 For instance, the United States planted 33.14 million hectares with transgenic maize seeds in 2018. In the same year, only two million hectares were planted with GMOs with a drought resistant gene stacked in their genome (ISAAA, 2018).

13 The term CRISPR (Clustered Regularly Interspaced Short Palindromic Repeats) refers to a natural defense system bacteria use against viral infections. When infected, bacteria synthesize enzymes (the most famous of them is called Cas9) which act as molecular scissors able to cut the virus DNA and store some fragments in their own genome. This procedure makes it possible that, in case of future infections, a bacteria recognizes the virus and destroys it (Pausch et al., 2020). The winners of the Nobel Prize for Chemistry in 2020, Jennifer Doudna and Emmanuelle Charpentier, showed that the Cas9 enzyme can be guided by the instructions of a messenger RNA molecule to recognize and cut DNA sequences of different types of organisms. With the cleavage, the gene is disabled, and, during its repair, the cut parts can be edited to correct genetic mutations or, in the specific case of the seed industry, to codify new useful functionalities in plants for agricultural activities (Zhang et al., 2019).
